# The familiarity of idiopathic scoliosis: statistical analysis and clinical considerations

**DOI:** 10.1186/1748-7161-7-S1-O73

**Published:** 2012-01-27

**Authors:** AG Aulisa, V Guzzanti, G Mastantuoni, M Giordano, A Poggiaroni, L Aulisa

**Affiliations:** 1 Children’s Hospital Bambino Gesù, Rome, Italy; 2 “A. Gemelli” Hospital, Università Cattolica del Sacro Cuore, Italy

## Background

To our knowledge the aetiology of idiopathic scoliosis is still unknown. It is likely caused by the interaction of multiple factors rather than by the action of a single responsible. The fact that idiopathic scoliosis is often seen in members of the same family has led researchers to investigate the role of genetic factors in the aetiology of this disease [1,2].

## Purpose of the study

The purpose of this study was to evaluate the impact of the familiarity of idiopathic scoliosis in a selected family sample.

## Materials and methods

The authors examined a family sample of 70 female patients with a relationship up to the third generation for a total of 2055 subjects. The parameters studied were: patient’s age at first observation, the type of curve and the mother’s and father’s age at the patient’s birth. The genealogy of the patients was investigated and related to the incidence of the disease.

## Results

The outcomes showed that 73% of the patients had an age between 12 and 15 years and that the thoracic localization of the curves was the most frequent. The 60% of the mothers had an age between 20-29 years and 57% of the patients were “first born”. The 5.8% of the brothers and the 12.7% of the sisters were affected by scoliosis. From the analysis of the total sample it is clear that in 53% of the families there is at least another scoliotic besides the patient, while in the remaining 47% she was the only one affected.

## Conclusions

The statistical analysis revealed three different types of transmission: multifactorial; autosomic dominant; autosomic recessive. Moreover female sex and firstborn resulted as risk factors of idiopathic scoliosis in the group of patients with multifactorial type of transmission.

## References

[B1] Merolli A Padua R Pitta L Aulisa AG Ceccarelli E Aulisa L A statistical analysis of the familial incidence of Idiopathic Scoliosis Stud Health Technol Inform 2002 88 37 15456002

[B2] Kouwenhoven JW Castelein RM The pathogenesis of adolescent idiopathic scoliosis: review of the literature Spine (Phila Pa 1976) 2008 33 26 2898 2908 10.1097/BRS.0b013e3181891751 19092622

